# Interactions Between Commensal Microbiota and Mucosal Immunity in Teleost Fish During Viral Infection With SVCV

**DOI:** 10.3389/fimmu.2021.654758

**Published:** 2021-04-07

**Authors:** Kai-Feng Meng, Li-Guo Ding, Sha Wu, Zheng-Ben Wu, Gao-Feng Cheng, Xue Zhai, Ru-Han Sun, Zhen Xu

**Affiliations:** ^1^ Department of Aquatic Animal Medicine, College of Fisheries, Huazhong Agricultural University, Wuhan, China; ^2^ Laboratory for Marine Biology and Biotechnology, Qingdao National Laboratory for Marine Science and Technology, Qingdao, China

**Keywords:** mucosal microbiota, immune responses, common carp (*Cyprinus carpio*), spring viremia of carp virus, 16S rRNA sequences

## Abstract

The mucosa of vertebrates is a particularly complex but dynamic environment in which the host constantly interacts with trillions of commensal microorganisms and pathogens. Although the internal and external mucosal microbiomes with immune defense of mammals have been well investigated, the relationship between mucosal microbes and their host’s immune responses has not been systematically understood in the early vertebrates. In this study, we compared the composition and distribution of mucosal microbiota in common carp (*Cyprinus carpio*), and found that there were significant differences of microbiota between in the internal (gut) and external mucosal (buccal mucosa, gills and skin) tissues. Next, we successfully constructed an infection model with spring viremia of carp virus (SVCV). Specifically, following viral infection, the immune and antiviral related genes showed different up-regulation in all selected mucosal tissues while significant morphological changes were only found in external tissues including buccal mucosa, gills and skin. Using 16S rRNA gene sequence, we revealed that the abundance of *Proteobacteria* in mucosal tissues including buccal mucosa, gills and gut showed increased trend after viral infection, whereas the abundance of *Fusobacteria* significantly decreased in gut. In addition, the loss of dominant commensal microorganisms and increased colonization of opportunistic bacteria were discovered in the mucosal surfaces indicating that a secondary bacterial infection might occur in these mucosal tissues after viral infection. Overall, our results firstly point out the distribution of internal and external mucosal microbiota and analyze the changes of mucosal microbiota in common carp after SVCV infection, which may indicated that the potential role of mucosal microbiota in the antiviral process in early vertebrates.

## Introduction

The mucosal surfaces of vertebrates constitute physical and chemical barriers that separate hosts from the external environment, and are inhabited by dense and complex populations of microorganism that play an essential role in digestion and nutrition, in addition to protecting hosts against pathogens and environmental insult (1–3). It has been proposed that the mucosal immune system in vertebrates may have evolved as a result of the complex symbiotic relationships between microbial communities and their host, which highlights the critical role of mucosal microbiota for maintaining the health of virtually all known vertebrates (4). Over the course of evolution and water-to-land transition in vertebrates, mucosal surfaces have undergone drastic changes that have resulted in different mucosal microbiota structures (2). Specifically, aquatic animals are continuously exposed to microbial-rich environments (freshwater or seawater) and may face a greater challenge coping with the complex microbial loads in their mucosal surfaces compared to land animals (5). As the outer mucosal tissues in teleost fish, the skin and gills have been extensively investigated and found that that they are largely dominated by obligate aerobes (6). However, skin and gills exhibit different microbial compositions. For instance, a study reported that fish skin were rich in Firmicutes and Actinobacteria, whereas the gills were dominated by Proteobacteria and Bacteroidetes which may be related to the gas exchange process of fish (2). In our previous study, we identified IgT-coated trout gills bacteria belonged to the Proteobacteria and Bacteroidetes phyla, suggesting that the members of these two phyla may have a protective role against pathogenic invasion (3). Additionally, several commensal microbiota isolates belonging to the Proteobacteria phylum in salmonid skin reportedly have an inhibitory effect on bacterial pathogen infection, further confirming that symbiotic bacteria may play a crucial role in fighting mucosal pathogens in teleost hosts (7). Interestingly, the buccal mucosa of teleost (i.e., the first tissue in the digestive tract that comes in contact with external stimuli) is a newly discovered mucosa-associated lymphoid tissue (MALT). Recent studies have determined that *Proteobacteria* was the most abundant phylum in teleost buccal mucosa; however, its abundance decreases significantly after infectious hematopoietic necrosis virus (IHNV) infection in trout (8–10). Unlike the microbial composition of skin and gills, the teleost gut is primarily colonized by aerobic, facultative anaerobic and obligate anaerobic bacteria (11). Importantly, Proteobacteria are the predominant gut microbiota in most marine and freshwater fish; however, these bacteria may become pathogenic under stressful conditions (10, 12–16). It is obvious that different mucosal tissues in teleost are inhabited by uniquely different microbial communities and proportions of specific bacteria (2, 17). However, although there is a topographical map of the microbiomes associated with pathogens or the environment (2, 18), the relationship between virus-mediated microbes and their hosts’ mucosal responses in external and internal mucosal surfaces remains uncharacterized.

This study focused on the common carp (Cyprinus carpio), one of the most widely farmed freshwater fish species worldwide, which is often challenged by the emergence of infectious diseases including bacterial, parasitic and viral pathogens (19, 20). Particularly, the spring viremia of carp virus (SVCV) poses a severe risk not only to carp health but also that of other aquatic animals (21). To better understand the microbial dynamic changes in different mucosal surfaces very early after viral infection, we successfully constructed an SVCV infection model where a less lethal concentration of SVCV was administered to common carp *via* injection, an easy, quick, and accurate immune method. Our results demonstrated that SVCV infection of the common carp elicited strong pathological changes and immune response both in external (buccal mucosa, skin, and gills) and internal (gut) mucosal tissues. Importantly, SVCV can cause microbial dysbiosis at the mucosal surface, leading to the invasion of opportunistic pathogens, suggesting that viral infection may be followed by secondary bacterial infection in the mucosal tissues. Moreover, we firstly compared the internal and external microbial structure during SVCV infection and provided a topographical map of the microbiome of a teleost species, thus highlighting the potential role of resident strains in aquatic viral disease control.

## Materials and Methods

### Fish Maintenance

The 5 months-old common carp (10–15 *g*) used in this experiment were obtained from a fish farm in Chongqing province, and maintained in aquarium tanks using a water recirculation system including thermostatic temperature control and extensive biofiltration. The fish were kept at 18°C for at least two weeks and fed with commercial carp pellets with a rate of 0.5–1% body weight twice a day (9:00 a.m. and 4:00 p.m.). The feeding was terminated 48 h prior to sacrifice both in control and infected groups. Before experimental infection, the fish were acclimatized to the water temperature by changing the water temperature from 18 to 12°C by 2°C per day for the SVCV infection. All animal procedures were approved by the Animal Experiment Committee of Huazhong Agricultural University and carried out according to the recommendations in the Guide for the Care and Use of Laboratory Animals of the Ministry of Science and Technology of China.

### Virus and Infection

In a 26°C incubator with 5% CO_2_, the cyprinus carpio epithelioma papillosum cyprini (EPC) cell line was maintained in minimum Eagle’s medium (MEM) supplemented with 10% fetal bovine serum (FBS) containing 1% Penicillin-Streptomycin Solution. The SVCV used in this study was gifted from Professor Xue-Qin Liu’s lab in the Huazhong Agricultural University and propagated in EPC cells until cytopathic effect (CPE) was observed, subsequently adjusted to 1 × 10^7^ pfu ml^−1^ in MEM and stored at −80°C until use. The methods used for SVCV infection were described previously by Wei X et al. (22) with slight modification. Briefly, fish were anaesthetized with methanesulfonate (MS-222) at a final concentration of 40 μg/ml and intraperitoneally (i.p.) injected with 100 μl of MEM containing SVCV. As the control group, fish were treated similarly and i.p. injected with 100 μl of MEM collected from non-infected cells. Then the fish were migrated into the aquarium containing new aquatic water.

### Sample Collection

After 4 days infection, the common carp were anesthetized with MS-222 for sampling. For histological and pathological studies, four different tissues (buccal mucosa, gills, skin, gut) of common carp were directly taken out from control fish and infected fish, then fixed immediately at 4% (v/v) neutral buffer paraformaldehyde for at least 24 h. For RNA extraction and quantitative real-time PCR (qRT-PCR), tissues including buccal mucosa, gills, skin, gut and spleen were collected in sterile micro-centrifuge tubes. For bacteria 16S rRNA gene sequencing, mucosa-associated bacteria were collected by scraping the mucosal layer with a sterile scalpel. Concretely, buccal upper mucosa was used for histological and pathological studies, quantitative real-time PCR and bacteria 16S rRNA gene sequencing. Gills taken from the second and third on the left and right gill arch were used for histological and pathological studies, quantitative real-time PCR and bacteria 16S rRNA gene sequencing. For skin, the skin on the back of the pectoral fin was sampled for histological and pathological studies; the skin on the back of the pectoral fin and below the dorsal fin was used for quantitative real-time PCR and bacteria 16S rRNA gene sequencing. For gut, after gently removing the contents, the foregut was used for histological and pathological studies, and the whole gut was used for quantitative real-time PCR and bacteria 16S rRNA gene sequencing. All tissues collected for RNA or 16S rRNA gene analysis were immediately frozen in liquid nitrogen and stored at -80°C for further study.

### Histology and Light Microscopy Studies

After fixed in 4% neutral formalin buffer, the buccal mucosa, gills, skin and gut were dehydrated in a graded ethanol series, washed with xylene, embedded in paraffin, and then sectioned into 5 μm pieces. The paraffin sections were stained with classic hematoxylin and eosin (H&E) as described previously (23). Images were acquired in microscope (Olympus, Japan) using the Axiovision software. By measuring the thickness of the epidermis, the microscopic pathological changes of the buccal mucosa and skin mucosa were evaluated. Similarly, the length–width ratios of the lamellae and villi were measured for evaluating microscopic pathological changes in gills and gut, respectively. The parameters of each image are measured by three different researchers and averaged to reduce random errors.

### RNA Isolation and Quantitative Real-Time PCR Analysis

Total RNA was extracted from fish different tissues (buccal mucosa, gills, skin, gut and spleen), which were homogenized in 1 mL TRIZol (Invitrogen) by shaking (60 HZ for 1 min) with steel beads. Equivalent amounts of the total RNA (1,000 *ng*) were used for cDNA synthesis with the SuperScript first-strand synthesis system for qPCR (YEASEN, China) in a 20 µl reaction volume. The synthesized cDNA was diluted 3 times and then was used as a template for qRT-PCR analysis. The qRT-PCR was performed on a qTOWER3G PCR system (Analytik Jena AG, Germany) by using the EvaGreen 2 × qPCR Master mix (YEASEN, China) as following conditions: 95°C for 5 min, followed by 40 cycles at 95°C for 10 s and at 58°C for 30 s. The change in transcription of genes was calculated as relative fold expression by the methods of 2^-ΔΔCt^ and 40S was used as control gene for normalization of expression. The results were obtained from three independent experiments and each was performed in triplicate.

### DNA Extraction and PCR Amplification

Microbial DNA was extracted from 32 samples using the OMEGA Soil DNA Kit (D5625-01) (Omega Bio-Tek, Norcross, GA, USA) according to manufacturer’s protocols. Subsequently we detected the DNA concentration and quality by NanoDrop ND-1000 spectrophotometer (Thermo Fisher Scientific, Waltham, MA, USA) and 2% agarose gel electrophoresis, respectively. The universal primer set 338F (5’-ACTCCTACGGGAGGCAGCA-3’) and 806R (5’-GGACTACHVGGGTWTCTAAT-3’) incorporated specific barcodes and was used for the amplification of the V3-V4 hypervariable region of bacterial 16S rRNA genes by thermocycler PCR system (GeneAmp 9700, ABI, USA). The PCR reactions were performed in triplicate 25 µl mixture containing 5 µl of buffer (5×), 0.25 μl of Fast pfu DNA Polymerase (5 U/μl), 2 μl (2.5 mM) of dNTPs, 1 μl (10 uM) of each Forward and Reverse primer, 1 μl of DNA Template, and 14.75 μl of ddH2O with the following condition: 5 min of denaturation at 98°C, 28 cycles of 30 s at 98°C, 30 s for annealing at 55°C, and 45 s for elongation at 72°C, and a final extension at 72°C for 5 min. The PCR amplicons were extracted from recycling of 2% agarose gel and further purified with Vazyme VAHTSTM DNA Clean Beads (Vazyme, Nanjing, China) and quantified using the Quant-iT PicoGreen dsDNA Assay Kit (Invitrogen, Carlsbad, CA, USA) according to the manufacturer’s protocol.

### Illumina MiSeq Sequencing and Analyses

Purified amplicons were pooled in equimolar and paired-end sequenced (2 × 300) on an Illumina MiSeq platform with MiSeq Reagent Kit v3 at Shanghai Personal Biotechnology Co., Ltd (Shanghai, China). Microbiome bioinformatics were performed with QIIME2 2019.4 (24) with slight modification according to the official tutorials (https://docs.qiime2.org/2019.4/tutorials/). Briefly, raw sequence data were demultiplexed using the demux plugin following by primers cutting with cutadapt plugin (25). Sequences were then quality filtered, denoised, merged and chimera removed using the DADA2 plugin (26). Alpha-diversity metrics (Chao1, Shannon, and Simpson), beta diversity metrics (weighted UniFrac) were estimated using the diversity plugin. Taxonomy was assigned to ASVs using the classify-sklearn naïve Bayes taxonomy classifier in feature-classifier plugin against the SILVA Release 132. For Lefse analysis, non-parametric factor Kruskal–Wallis rank sum test is applied for determining the species that showed significant difference in abundance. By linear discrimination analysis (LDA), the effect of the different species was estimated.

### Standard Curve for Spring Viremia of Carp Virus

For standard curve, the PCR products of SVCV were inserted in pMD 19-T vector and recombined with DH5α *Escherichia coli* cells. Plasmid DNA was isolated from an overnight selective culture using HiPure Plasmid Micro Kit (OMEGA). For estimation of plasmid copy number, the following equation was used:$\text{copies}/\mu \text{l}=\frac{6.023\times 10^{23}\times 4.05\times 10^{-9}}{2916\times 660}$, where Avogadro number = 6.023×10^23^ molecules/mol; plasmid concentration =4.05×10^-3^ μg/μl; recombinant plasmids = 2916 bp and average MW of a DNA molecule = 660 g/mol. The recombinant plasmids diluted 10 times continuously (a total of 7 gradients of 1.27 × 10^8^ copies/μl ~1.27 × 10^2^ copies/μl) were used as the standard positive template. The standard curve was shown in **Supplementary Figure 3**, and the Ct values of the samples were extrapolated into the standard curve to calculate the copy number.

### Statistical Analysis

An unpaired Student’s *t*-test (Prism version 6.0; GraphPad) was used for gene expression and histology data analysis. For 16S analysis, Mann-Whitney test was used to evaluate the differences between control and infection groups. *P*-values of 0.05 or less were considered statistically significant.

## Results

### Microbiome Signatures Across Different Mucosal Sites in Common Carp

In this study, four mucosal tissues including external buccal mucosa (BM), gills, skin and internal gut mucosa were collected, and the abundance of their microbial communities was assessed using 16S rRNA sequencing with the Illumina MiSeq platform. We obtained a total of 40,425,311 sequences from the original samples of control and infected fish, which were further filtered down to 3,387,816 merged sequences after removal of the samples with the threshold. Afterward, the sequences were divided into unique OTUs at the 97% level using the DADA2 plugin and further clustered into 21,780 distinct OTUs for downstream analysis. Concretely, the BM, gills, skin, and gut had a total of 23, 25, 22, and 24 phyla, respectively. Analysis at the genus level elucidated 160, 173, 156, and 150 genera in BM, gills, skin, and gut, respectively.

**Figure 1:**
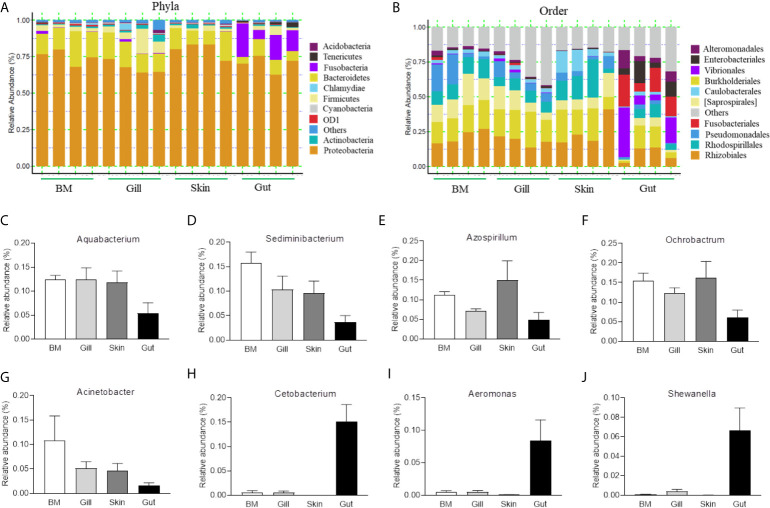
Composition and distribution of the bacterial microbiome in common carp at different mucosal sites. **(A)** Composition and relative abundance of the dominant bacterial taxa in common carp samples (BM, gills, skin, and gut) at the phylum level. **(B)** Composition and relative abundance of the dominant bacterial taxa in common carp samples (BM, gills, skin, and gut) at the order level. **(C–I)** The distribution of several representative dominant bacteria in various mucosal tissues (BM, gills, skin, and gut) in common carp including *Aquabacterium* **(C)**, *Sedminibacterium* **(D)**, *Azospirillum* **(E)**, *Ochrobactrum* **(F)**, *Acinetobacter* **(G)**, *Cetobacterium* **(H)**, *Areomonas* **(I)**, and *Shewanella* **(J)**, BM, buccal mucosa; Data are representative of 4 individuals (mean ± SEM).

To further compare the microbial composition and distribution in different mucosal sites of common carp, the external (BM, gills, skin) and internal (gut) tissues were characterized at the phylum and order levels (**Figures 1A, B**). Similar to previous studies, *Proteobacteria* was the most predominant phylum both in internal and external mucosal sites of common carp, accounting for a large proportion in each site (BM, 74.9%; skin, 67.6%; gills, 79.8%; gut, 70.2%). After *Proteobacteria*, *Bacteroidetes* and *Fusobacteria* accounted for 15.4% and 15.1% in external and internal mucosal sites, respectively. Interestingly, we found that the microbial composition of the external and internal mucosa was also significantly different at the order level. For example, the aerobic microorganisms *Rhizobiale*s, *Burkholderiales*, and *Saprospirales* made up the majority of the external microorganisms (18.4%, 16.5%, and 10.0%, respectively). However, the gut microbiota was mainly composed of anaerobes and facultative anaerobes such as *Vibrionales*, *Fusobacteriales*, *Enterobacteriales*, and *Alteromonadales* (16.1%, 15.1%, 8.7%, and 6.9%, respectively). Additionally, upon analyzing the bacterial OTUs at the genus level, we identified the differences in microbial communities between external and internal mucosa including beneficial bacteria such as *Aquabacterium*, *Sediminibacterium*, *Azospirillum*, and *Cetobacterium*, as well as disease-causing taxa such as *Ochrobactrum*, *Acinetobacter*, *Aeromonas*, and *Shewanella* (**Figures 1C–J**). Importantly, the proportion of *Aquabacterium*, *Sediminibacterium*, *Azospirillum*, *Ochrobactrum*, and *Acinetobacter* in external mucosal sites were homogeneous and undifferentiated, whereas the gut exhibited a ~2-, and 3-fold decrease in the abundance of these microbial communities. In contrast, the relative abundance of *Cetobacterium*, *Aeromonas*, and *Shewanella* in the internal gut mucosa was much higher than in the external mucosal sites. Overall, our findings highlighted the variations in the composition and distribution of external and internal mucosal microbial communities in common carp.

### SVCV Infection Induced Morphological Changes and Immune Genes Expression in Common Carp

**Figure 2:**
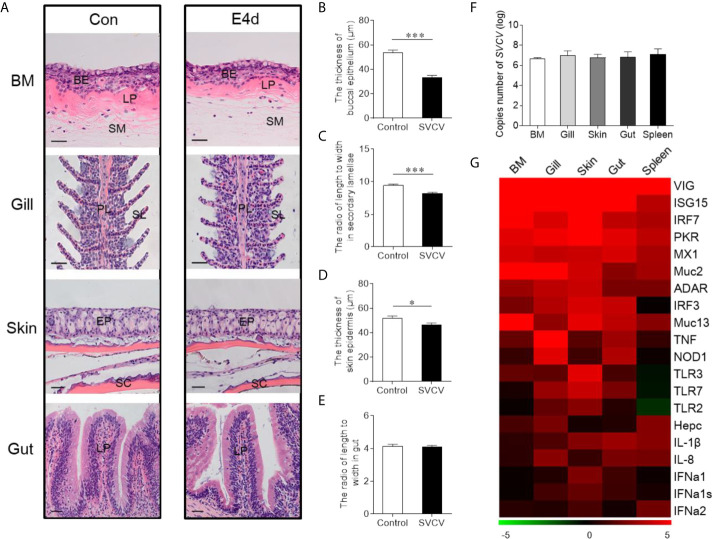
Pathological changes and immune response in mucosal tissues of common carp following SVCV infection. **(A)** Histological examination of the mucosal tissues (including BM, gills, skin, and gut) from control fish and experimental fish infected with SVCV after 4 days (*n* = 6 fish per group). **(B)** The thickness of BM epidermis in control and infected fish (*n* = 6 fish per group). **(C)** The length-width ratio of secondary lamellae in control and infected fish (*n* = 6 fish per group). **(D)** The thickness of skin epidermis in control and infected fish (*n* = 6 fish per group). **(E)** The length-width ratio of gut villus in control and infected fish (*n* = 6 fish per group). **(F)** The loads of SVCV in different tissues (BM, gills, skin, gut, and spleen) at 4 days after infection (*n* = 9 fish per group). Data are representative of three independent experiments (mean ± SEM). **(G)** Heat map illustrates results from quantitative real-time PCR of mRNAs for immune-related genes in virus-challenged fish vs. control group measured at 4 days following with SVCV in the BM, gills, skin, gut, and spleen of common carp (*n* = 6 fish per group). Color value: log2 (fold change). BM, buccal mucosa; BE, buccal epithelium; SM, submucosa; PL, primary lamellae; SL, secondary lamellae; EP, epidermis; SC, scales; LP, lamina propria; Scale bars, 20 µm. Control vs. Infected: *P < 0.05, ***P < 0.001, unpaired Student’s *t*-test. Data are representative of three different independent experiments (mean ± SEM).

Given the observed variations in the composition and distribution of internal and external mucosal microorganisms, we then sought to characterize the effect of pathogen invasion on mucosal tissues. Here, we constructed an infection model with SVCV, which was harvested by proliferation in EPC cells (**Supplementary Figure 1A**). As expected, typical symptoms such as proptosis in the eyes, hyperemia in the fins, and swelling in the anus were observed in the infected group (**Supplementary Figure 1B**). In contrast, the control group did not exhibit any of these clinical signs throughout the experimental period. We then collected the samples including BM, skin, gills, gut, and spleen after SVCV infection, and detected high SVCV expression in most tissues at 4 days post-infection (**Supplementary Figure 2**). Hematoxylin and eosin (H&E) staining was then conducted to evaluate the morphological changes in mucosal tissues of common carp after SVCV infection. These histological analyses revealed that the thickness of the epidermis (EP) in the buccal mucosa and skin had significantly contracted at 4 days post-infection compared to that of control carp (**Figures 2A, B,** and **D**). Moreover, significant changes in infected gills were also observed, as evidenced by wider and shorter secondary lamellae (**Figures 2A, C**). However, no pathological changes were detected in the internal gut mucosa, which was consistent with a previous study in which no conspicuous changes were found in the gut of rainbow trout after virus infection compared to control fish (10) (**Figures 2A, E**). Interestingly, high copy numbers of SVCV were detected in both external (buccal mucosa, skin, and gills) and internal (gut) mucosa. As expected, SVCV was detected frequently in the spleen (**Figure 2F**). Overall, these results demonstrated that SVCV successfully invaded investigated tissues of common carp and induced significant morphological changes in the external mucosa.

To gain insights into the kinetics of the immune responses following SVCV infection, the expression of 20 antiviral and immune-related genes was quantified at 4 days post-infection in external (buccal mucosa, skin, and gills) and internal (gut) mucosal tissues, as well as spleen tissues *via* RT-qPCR, including pro-inflammatory cytokines genes (interleukin 1β and interleukin 8), toll-like receptors (TLR2, TLR3, and TLR7), interferon (IFNa1, IFNa1s, and IFNa2) and interferon regulator factor (IRF3 and IRF7), antimicrobial peptides (Hepcidin), mucins (Muc2 and Muc13), innate immune genes (TNF and NOD1) and antiviral genes (ISG15, Mx1, VIG, protein kinase R (PKR), ADAR) (**Figure 2G**; the primers used in this study are shown in **Supplementary Table 1**). Strong antiviral responses were detected in both mucosal tissues (BM, gills, skin, and gut) and spleen tissue after SVCV infection, which further suggested that the common carp was successfully invaded by SVCV and activated the antiviral pathway. Moreover, the expression of immune genes was also detected in external and internal mucosal tissues, indicating that innate immunity was involved in the antiviral process. Interestingly, the relative expression level of antiviral response genes was similar to that of immune-related genes in mucosal tissues such as IRF7, which is a master transcriptional factor that regulates IFN gene induction and innate immune response after virus infection. Similarly, IFN, a critical secreted mediator of the innate immune response, also exhibited the same expression pattern in both mucosal tissues and the spleen. Additionally, we found that the up-regulated expression of immune genes in the spleen was lower than that in mucosal tissues, which indicated that mucosal tissues may play a more important role in the early stages of viral infection than the spleen.

### Changes in the Microbial Distribution of Mucosal Tissues After SVCV Infection

**Figure 3:**
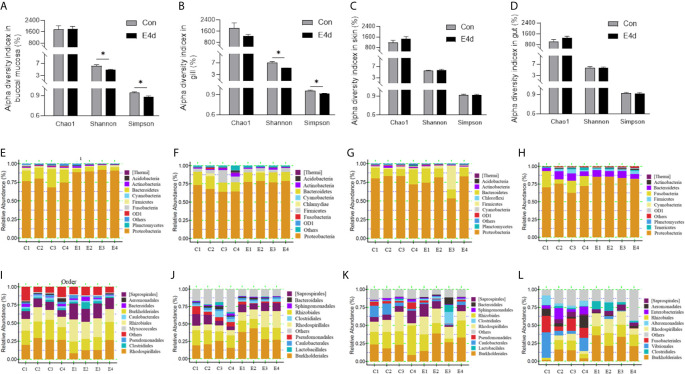
Microbiota community changes in mucosal tissues of common carp in response to SVCV infection. **(A–D)** Alpha diversity of bacterial community in common carp BM **(A)**, gills **(B)**, skin **(C)**, and gut **(D)** from control and infected groups (*n* = 4 fish per group). Richness and diversity of the bacterial community was measured using Chao1 mean and Shannon index, respectively. Error bars represent standard error of mean (SEM). Kruskal-Wallis test was conducted between groups with significance level. Control vs. Infection: *P < 0.05. **(E–H)** Bar chart of the relative abundance of phylum present at BM **(E)**, gills **(F)**, skin **(G)**, and gut **(H)** from control fish and experimental fish infected with SVCV after 4 days. **(I–L)** Bar chart of the relative abundance of order present at BM **(I)**, gills **(J)**, skin **(K)**, and gut **(L)** from control fish and experimental fish infected with SVCV after 4 days. BM, buccal mucosa; E4d, 4 days after SVCV infection.

Next, we calculated the differences in the microbial abundance and community diversity in the mucosal tissues between infected and control groups. Interestingly, the Shannon diversity index (a metric that weights the numbers of species by their relative evenness data) and the Simpson diversity index (a metric that weights species diversity by their richness and evenness) in BM and gills decreased significantly after SVCV infection compared to the control group (**Figures 3A, B**). However, the Chao1 index (a metric used to estimate microbial richness) did not change significantly in any of the selected mucosal sites including BM, gills, skin, and gut (**Figures 3A–D**). To further analyze the microbial composition changes in the external and internal mucosal sites, the microbial sequences from the control and infected fish were classified by phylum, class, order, family, and genus. At the phylum level, the abundance of *Proteobacteria* increased significantly post SVCV infection in BM (90.3% in the infected group versus 74.9% in the controls), gills (78.2% in the infected group versus 67.7% in the controls), and gut (83.8% in the infected group versus 70.2% in the controls) but decreased in the skin (73.2% in the infected group versus 79.8% in the controls). Notably, the abundance of *Fusobacteria* in the infected common carp gut decreased significantly compared to control fish (0.4% in the infected group versus 15.1% in the controls) (**Figures 3E–H**). At the order level, although the changes in the microbial composition of external mucosal sites were moderate, we observed an increasing trend in pathogenic bacteria and a decrease in beneficial bacteria (**Figures 3I–K**). Specifically, the abundance of the pathogenic bacteria *Burkholderiales* increased in the BM (~2.4-fold), gills (~1.7-fold), and skin (~1.5-fold), whereas the abundance of the beneficial orders *Bacteroidales* in gills and *Clostridiales* in BM and gills decreased upon viral challenge (~1.5-fold, ~2.0-fold, and ~1.5-fold, respectively). Although no significant signs of tissue damage were observed in the gut after SVCV infection, this tissue exhibited dramatic changes in bacterial abundance (**Figures 2E** and **3L**). For instance, the abundance of *Rhodospirillales*, *Burkholderiales*, and *Rhizobiales* increased (~3.2-fold, ~3.0-fold, and ~1.8-fold, respectively), whereas the abundance of *Enterobacteriales*, *Fusobacteriales*, *Vibrionales*, and *Alteromonadales* decreased (~48.8-fold, ~41.9-fold, ~12.9-fold, and ~6.9-fold, respectively). In general, SVCV invasion disrupts microbial homeostasis both in the external and internal mucosal tissues of common carp, which may lead to opportunistic pathogen invasion and secondary infection.

### SVCV Infection Led to Significant Alterations in the Microbial Community of Different Common Carp Mucosal Tissues

**Figure 4:**
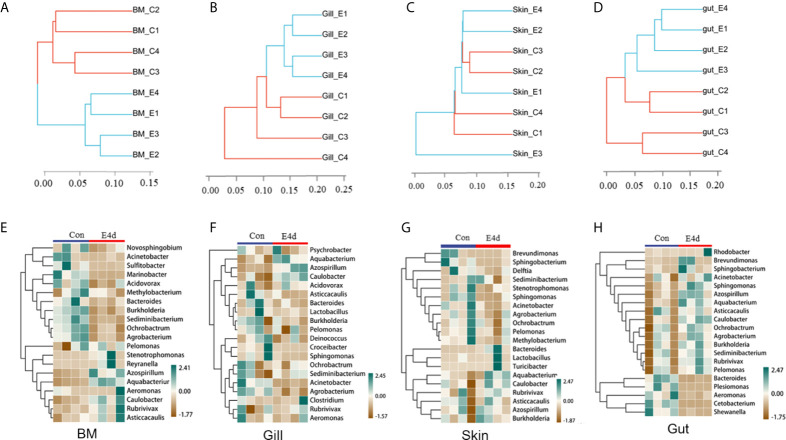
The diversification of microbiota community at genus level in common carp mucosal tissues after SVCV infection. **(A–D)** Hierarchical cluster analysis of Weighted-unifrac distances generated from BM **(A)**, gills **(B)**, skin **(C)**, and gut **(D)** in control group and SVCV-challenged group. **(E–H)** Heat map showing the hierarchical clustering results for the abundance of bacterial genera in BM **(E)**, gills **(F)**, skin **(G)**, and gut **(H)** from SVCV-challenged and control fish. BM, buccal mucosa; E4d, 4 days after SVCV infection. Pheatmap package of R (version 3.4.4) was used to picture heat maps, Pearson correlation was carried out and Weighted-unifrac method was used to cluster the relative abundance values. The relative abundance values were scaled in raw.

Based on the WPGMA (Weighted pair group method with arithmetic mean) algorithm, we performed a hierarchical clustering tree, and found that the microbial communities in the BM, gills, and gut tissues of control and infected groups were clustered into two distinct groups (**Figures 4A, B,** and **D**). However, the OTU clustering in the skin from control and infected fish was dispersed (**Figure 4C**), which may be caused by individual differences and sampling conditions. Details about the changes of the microbial community at the genus level were shown with heatmaps including the top 20 bacteria from control and infected groups (**Figures 4E–H**). Interestingly, the abundances of *Azospirillum*, *Aquabacterium*, and *Caulobacter* were much higher in all of the mucosal tissues of the infected group compared to the controls. Additionally, the abundances of *Acinetobacter*, *Ochrobacterium*, and *Agrobacterium* decreased in the external mucosal tissues but increased in the internal gut mucosa. Moreover, we observed a decrease in the abundance of *Plesiomonas*, *Cetobacterium*, and *Shewanella*, which were uniquely dominant in the gut compared to the control group.

**Figure 5:**
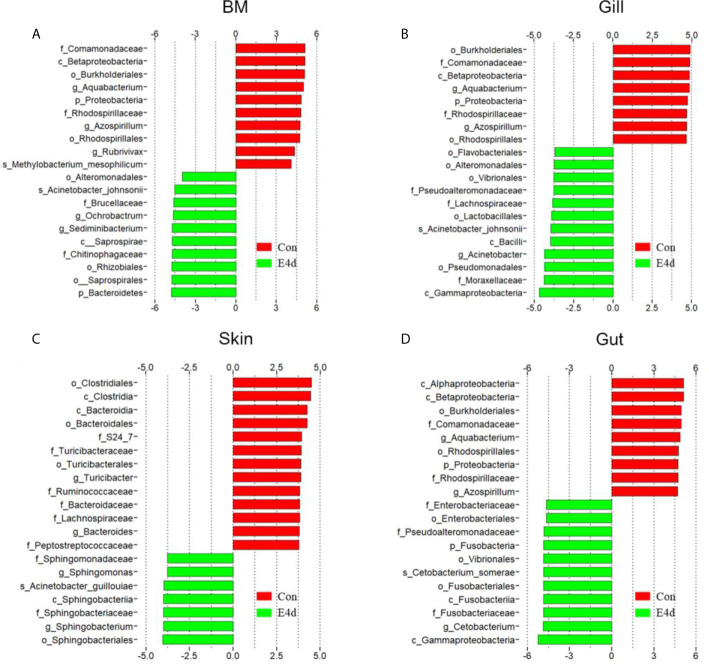
Description of biomarkers that were significantly different between control and infection groups in common carp BM **(A)**, gills **(B)**, skin **(C)**, and gut **(D)**. E4d, 4 days after SVCV infection.

**Figure 6:**
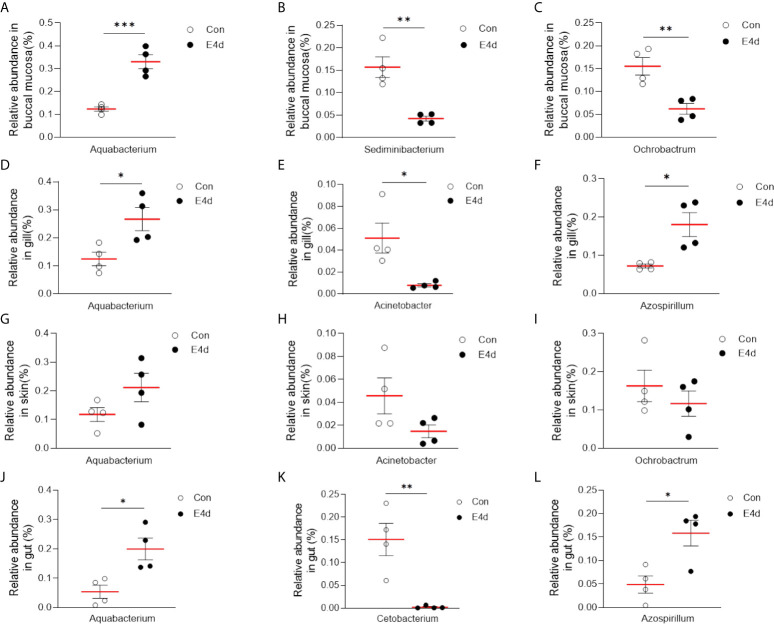
SVCV infection results in microbial composition changes in common carp BM **(A–C)**, gills **(D–F)**, skin **(G–I)**, and gut **(J–L)**. *P < 0.05, **P < 0.01, ***P < 0.001, unpaired Student’s *t*-test.

LDA effect size (LEfSe) analysis was used to further explore the changes in microbial composition in different common carp tissues after SVCV infection. Here, we identified significant decreases in the abundance of *Aquabacterium* and *Azospirillum* in BM, gills, and gut (~5.0-fold and ~5.1-fold, ~4.8-fold and ~4.9-fold, ~4.8-fold and ~4.9-fold, respectively). In contrast, *Ochrobactrum, Acinetobacter*, and *Cetobacterium* decreased more than ~4-fold in BM, gills, and gut after SVCV invasion (**Figures 5A, B**, and **D**). Moreover, the abundance of *Sediminibacterium* decreased by ~4.7-fold in the BM. Interestingly, the microbial changes of the skin were inconsistent with other tested mucosal tissues (**Figure 5C**). For instance, the abundances of *Turicibacter* and *Bacteroides* in the skin were significantly increased at the expense of losses in *Sphingobacterium* and *Sphingomonas* after SVCV infection. Additionally, we performed scatter diagrams to illustrate the changes in dominant bacteria both in internal and external mucosal tissues (**Figure 6**). Interestingly, although no significant differences were detected in the skin of common carp, the abundance of *Aquabacterium* increased in BM, gills, skin, and gut (**Figures 6A, D, G**, and **J**). However, *Sediminibacterium* and *Ochrobactrum* were markedly decreased in the BM as a result of SVCV infection (**Figure 5A** and **Figures 6B, C**). Furthermore, a significant decrease in *Acinetobacter* and an increase in *Azospirillum* were observed in gills tissues at 4 days post-infection compared to the control group (**Figures 6H, I**). A moderate decrease in *Acinetobacter* and *Ochrobactrum* was also observed in the skin (**Figures 6E, F**), whereas a decrease in *Cetobacterium* and an increase in *Azospirillum* were detected in the gut (**Figures 6K, L**).

## Discussion

The microorganisms that inhabit the mucosal surfaces of vertebrates play critical roles in the development, homeostasis, and immune function of mucosal tissues. However, imbalances in the microbiota of susceptible hosts may lead to opportunistic pathogenic invasion and a multitude of immune-mediated disorders (27). Many studies on mammalian mucosal microbiota have identified distinct microbial communities in the mouth, skin, gut, and vaginal cavity (17). In teleost, although some studies have characterized the changes in the microbial composition of mucosal tissues in response to pathogen infection or environmental changes, very few studies have assessed the effect of viral infection on the internal and external mucosal microbiomes of aquatic animals. Therefore, our study sought to compare the microbial composition of external (BM, gills, and skin) and internal (gut) mucosal tissues in common carp and analyzed the changes of microorganism and immune response after SVCV infection.

16S rRNA sequencing analyses revealed differences in the bacterial community composition of different mucosal tissues including BM, gills, skin, and gut. Similar to rainbow trout (2, 10, 28), southern catfish (18), and zebrafish (29), *Proteobacteria* and *Bacteriodetes* accounted for a large proportion of both the internal and external mucosal microbiota of common carp. Previous studies have linked *Proteobacteria* and Bacteroidetes to inflammation and metabolic diseases, respectively, thus acting as “microbial signatures” of disease (30–32). However, the abundance of *Fusobacteria* in the gut was significantly higher than that in external mucosal tissues, which was attributed to the unique characteristics of the gut environment (33). Similarly, we also found that the bacterial composition of internal (gut) mucosa at the order level was markedly different from that of external tissues, as shown in previous studies in other species (17). Particularly, the abundance of *Fusobacteriales*, *Enterbacteriales*, and *Vibrionales* were much higher in the gut compared to the external mucosa, suggesting that these bacterial taxa might be related to nutrition, metabolism, and immune function in the gut according to the previous studies (33–35). Therefore, the bacterial composition observed in external mucosal sites may be a reflection of niche and environmental diversity, whereas the gut may offer more stable habitats that shape specialized microbial communities.

To evaluate the dynamic changes of the immune responses and microbial composition in response to viral infection, an SVCV infection model was established in common carp. We found that SVCV could successfully invade investigated tissues of common carp and caused typical clinical symptoms (e.g., proptosis in the eyes, hyperemia in the fins, and anal swelling). Moreover, significant morphological changes were detected in external tissues, including lamellae epithelium thickening or mucosal epithelium thinning, which may be caused by the loss of epithelial mucous cells while released mucus in response to viral invasion according to previous study (10). However, compared to control fish, no pathological changes were detected in the gut, which was consistent with previous study (10). We speculated that the gut would be the habitat of the special microbial community, which ensured the integrity of the gut in the process of antiviral infection (20, 36). The morphological changes caused by SVCV also likely led to changes in the expression of immune genes in the corresponding tissues. Previous studies have shown that virus infection often leads to the activation of innate immune signal pathways (37). In our study, a strong immune response was observed both in the internal and external mucosa of infected common carp. As key components of intercellular signal transmission and regulation, the expression of cytokines including inflammatory factors (IL-1β and IL-8) and toll-like receptors (TLR2, TLR3, and TLR7) increased significantly after viral infection (22, 38). According to previous studies, IFNs are secreted mediators that play a fundamental role in the innate immune response against viruses in all vertebrate classes (39). Our study detected a high expression of interferon-related genes in mucosal tissues including interferons (IFNa1, IFNa1s, and IFNa2) and interferon regulator factors (IRF3 and IRF7). Moreover, innate immune genes such as TNF and NOD1 also been detected in all selected mucosal tissues, further suggesting that innate immunity was involved in the antiviral process (40). More importantly, the mRNA expression of antiviral genes such as Vig1, ISG15, Adar, PKR, and Mx1 was upregulated in infected individuals, suggesting that SVCV activated the antiviral pathway in common carp (21, 22, 41). As an antimicrobial peptide widely distributed in teleost, hepcidin plays an important role against microbial invasion in the innate immune system (42). In our study, the expression of hepcidin increased significantly after SVCV infection, indicating that viral infection may cause antimicrobial immune responses in common carp. Moreover, as matrix containing a diverse range of antimicrobial molecules, mucin expression may also favor the colonization of mucosal surfaces with facultative bacterial pathogens in common carp according to previous study (20). Mucins such as Muc2 and Muc13 also were detected in all selected tissues in our study, further suggesting that SVCV invasion may lead to secondary infection. To our surprise, consistent with previous studies, strong immune responses were detected in the gut after virus infection though no pathological changes were identified (10). Importantly, we found that the expression levels of immune response genes in the spleen were lower than those in mucosal tissues, which may indicate that the spleen of teleost exhibits a delayed immune response to pathogen invasion (28).

Our study firstly characterized the changes in bacterial community in common carp mucosal tissues after SVCV infection and found that there was a significant change in bacterial community species, albeit without significant changes in microbial diversity. As mentioned above, previous studies on mammals have proposed that an increased prevalence of the bacterial phylum Proteobacteria could be used as a marker of unstable microbial structure and may constitute a potential criterion for disease diagnosis (30–32). In our study, the relative abundance of *Proteobacteria* in the BM, gills, and gut of infected common carp was significantly higher than that of the control group, which confirms the potential diagnostic value of this signature in teleost. However, consistent with previous studies on rainbow trout, SVCV infection decreased the relative abundance of *Proteobacteria* in the skin (28). Thus, based on the aforementioned studies, we speculated that pathogens may similarly impact fish microbiota diversity regardless of species. In our study, almost all Fusobacteria detected in fish gut mucosa belonged to the *Fusobacteriales*, which has been linked to host nutrition. Moreover, we found that the relative abundance of *Fusobacteriales* in common carp gut was significantly lower after SVCV infection, suggesting that the changes in gut microbial communities caused by SVCV infection may alter nutrient absorption capacity in common carp intestines.

Based on the changes in the bacterial communities of mucosal tissues at the phylum and order levels, our study used LEfSe analysis to identify the top 20 bacteria that underwent significant changes in each mucosal tissue. Interestingly, the abundance of Bacteroidetes increased in the skin but decreased in the BM, gills, and gut after SVCV infection. Studies have shown that Bacteroides members may encode a proportionally high number of carbohydrate-active enzymes (CAZymes; e.g., glycoside hydrolases and polysaccharide lyases) that enable the use of both dietary and host mucosal glycans (36), which implied that nutritional and metabolic processes in the gut may have been affected by SVCV infection. Importantly, previous studies have demonstrated that Bacteroides could activate intestinal dendritic cells (DCs) in the human gut, thus inducing plasma cells in the intestinal mucosa to express secretory IgA (sIgA) to coat the surface of gut microbiota (43). It is reasonable to extrapolate that Bacteroides in teleost may also play the same role. Unlike Bacteroides significantly increased abundance only in the skin, we found that the abundance of *Aquabacterium* strikingly increased in BM, gills, skin, and gut. Moreover, our previous study also showed that the abundance of *Aquabacterium* increased significantly and then decreased slightly after *Ichthyophthirius multifiliis* (Ich) infection (28). Interestingly, Ich infection led to increased colonization of opportunistic bacteria, which inevitably affected the colonization of symbiotic bacteria. Therefore, these results indicated that *Aquabacterium* may be a symbiotic bacterium that plays an important role after SVCV infection. In contrast, the abundance of Burkholderia, a group of bacteria that is considered potentially pathogenic in humans and insects (44), was markedly increased in the gut mucosa after SVCV infection, suggesting that viral infection may facilitate colonization by opportunistic bacteria.

**Figure 7:**
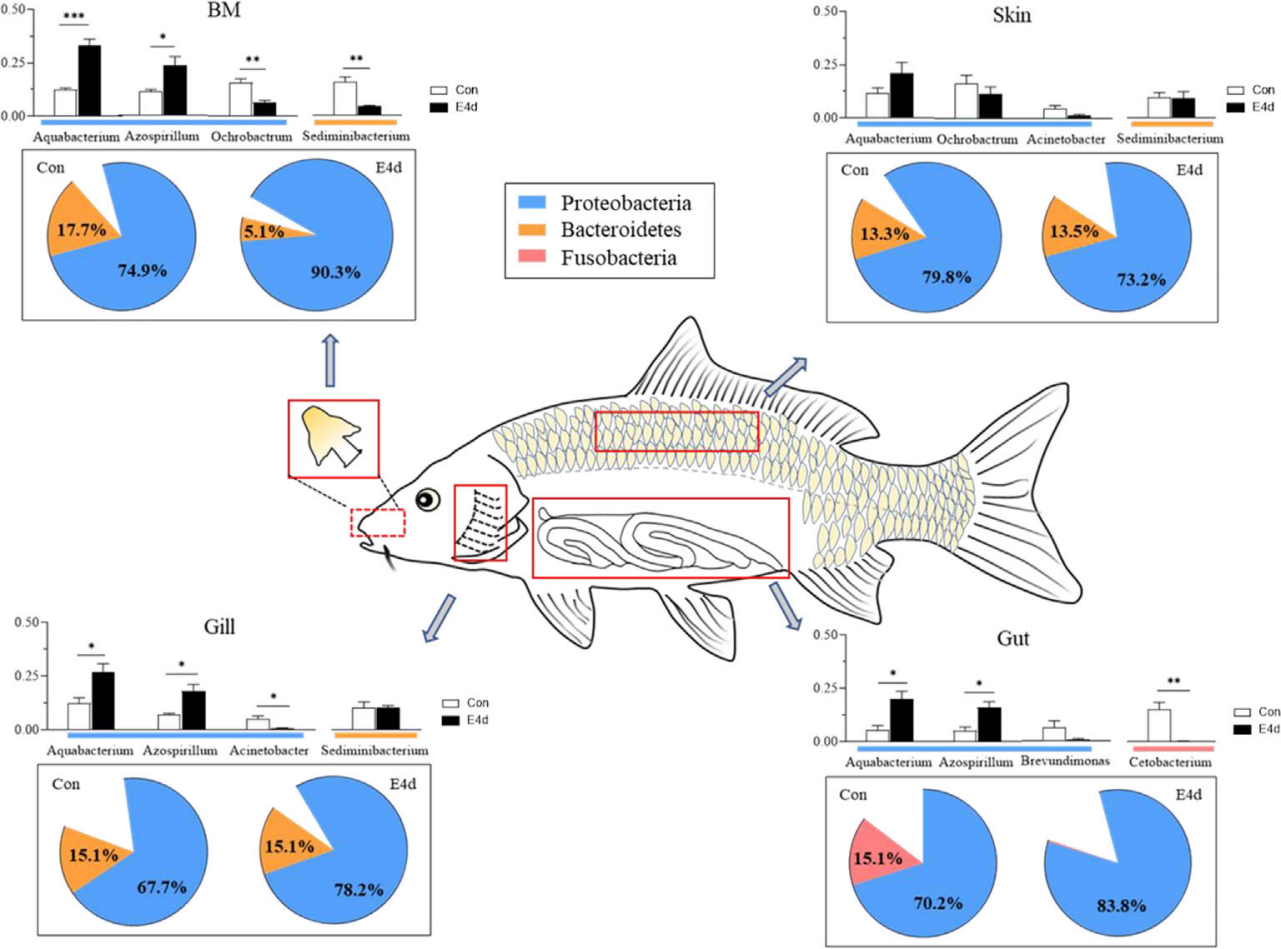
A map of microbiome community compositions at different mucosal sites (BM, gills, skin, and gut) in common carp after SVCV infection. The pie charts represent the microbial changes at the phylum level following SVCV infection. The histograms represent the changes of dominant bacteria abundance at the genus level following SVCV infection. Red rectangles show the sampling scheme used for 16S sequencing. *P < 0.05, **P < 0.01, ***P < 0.001, unpaired Student’s *t*-test. E4d, 4 days after SVCV infection.

In general, we found that Proteobacteria was the dominant microbial community in both external (BM, gills, and skin) and internal (gut) tissues of common carp, with *Fusobacteria* also accounting for a large proportion of the gut microbiota. After SVCV infection, the mucosal tissues exhibited a strong antiviral response, particularly in the external mucosa, and mucosal microorganisms also exhibited significant changes. The abundance of *Proteobacteria* in mucosal tissues including BM, gills, and gut exhibited an increasing trend after viral infection, whereas the abundance of *Fusobacteria* significantly decreased in the gut (**Figure 7**). More importantly, our study is the first to demonstrate that SVCV infection disrupts the microbial homeostasis of both external and internal mucosal tissues in common carp, which may lead to opportunistic pathogen invasion and secondary infection.

## Data Availability Statement

The raw data supporting the conclusions of this article will be made available by the authors, without undue reservation.

## Ethics Statement

The animal study was reviewed and approved by The Animal Experiment Committee of Huazhong Agricultural University.

## Author Contributions

K-FM performed most of the experiments and wrote the manuscript. K-FM and L-GD analyzed the data. SW, Z-BW, G-FC, XZ, and R-HS helped with most of the experiments. ZX designed the experiments and revised the manuscript. All authors contributed to the article and approved the submitted version.

## Funding

This work was supported by granted from National Key Research and Development Program of China (2018YFD0900400) and the National Natural Science Foundation of China (32073001).

## Conflict of Interest 

The authors declare that the research was conducted in the absence of any commercial or financial relationships that could be construed as a potential conflict of interest.
