# Supplementary material for: Interactions Between Commensal Microbiota and Mucosal Immunity in Teleost Fish During Viral Infection With SVCV
Source: Front Immunol. 2021 Apr 7;12:654758. doi: 10.3389/fimmu.2021.654758 (PMC8058427; doi:10.3389/fimmu.2021.654758)
Supplement: Supplementary file 1 [file Presentation_1.pdf]

Interactions between commensal microbiota and mucosal immunity in teleost fish during viral infection with SVCV

Kai-Feng Meng[1], Li-Guo Ding[1], Sha Wu[1], Zheng-Ben Wu[1], Gao-Feng Cheng[1], Xue Zhai[1], Ru-Han Sun[1] and Zhen Xu[1,2*]

[1]Department of Aquatic Animal Medicine, College of Fisheries, Huazhong Agricultural University, Wuhan, Hubei 430070, China

[2]Laboratory for Marine Biology and Biotechnology, Qingdao National Laboratory for Marine Science and Technology, Qingdao, Shandong 266071, China

[*]Corresponding Author: zhenxu@mail.hzau.edu.cn.

Supplementary materials

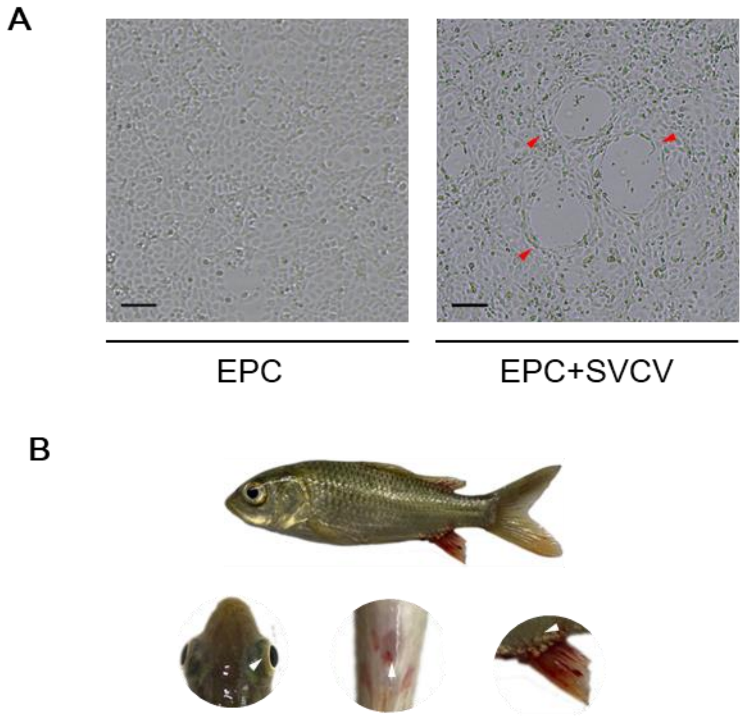

FIGURE S1 | Cytopathic and clinical symptoms in EPC and common carp caused by SVCV. **(A)** Normal EPC (left) and EPC infected with SVCV (right), scale bars, 100 μm, the red arrows represent CPE. **(B)** Typical symptoms such as proptosis in the eyes (left), swelling in the anus (middle), and hyperemia in the fins (right) in the infected common carp. The white arrows represent clinical symptoms.

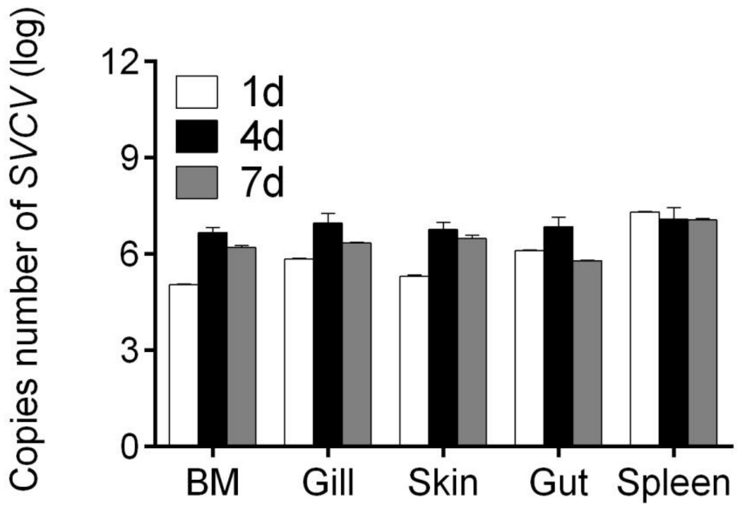

FIGURE S2 | Histogram demonstrated the loads of SVCV in different tissues (BM, gills, skin, gut, and spleen) at 1, 4, and 7 days after infection (*n* = 9 fish per group).

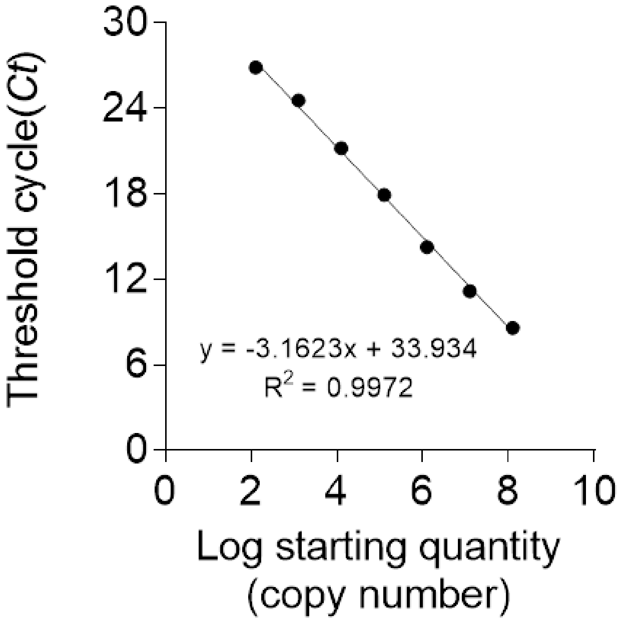

FIGURE S3 | Standard curve for SVCV load used in this study.

TABLE S1 | Primers used in this study.

Gene	GenBank accession no.	Primer Sequence (5’-3’)	Tm (◦C)	Amplicon Length (bp)
**REFERENCE GENE**				
40S	AB_012087.1	F: CCGTGGGTGACATCGTTACA<br>R: TCAGGACATTGAACCTCACTGTCT	58	69
**SPRING VIREMIA OF CARP VIRUS**				
SVCV-N	DQ_491000.1	F: CTCTGCCAAATCACCATACTC<br>R: GCGGTTTTCTGTATGTGTCTC	58	224
**ANTIVIRTAL GENES**				
Vig 1	JX_131617.1	F: CGCACCAAAGAGCAGAAAGA<br>R: AATGGGCAAGACGAAAGAGG	58	138
ISG 15	KP_115358.1	F:AAGCCATATTCAGCGAAGC<br>R:AACCGTTATCGGCAGACAG	58	185
PKR	EX_880666.1	F:CCAACATCGTCCGCTACTACTC<br>R:GCGTGTCTCCCTCACAAAG	58	147
MX1	KP_115357.1	F: GAACTTCGGGAAGAATTTGC<br>R: ATGAATCCTGGAAGCCCTC	58	131
ADAR	EC_392392.1	F:GCAGGACGAGGTGTCAGAG<br>R:GGCAAAGGGAGCATAACTTC	58	200
**IMMUNE RELATED GENES**				
Interleukin 1β	AB_010701.1	F: CAGAGCAACAAACTAAGTGACGAG<br>R: ACCATCTAACTGGGTACAAGCAAG	58	189
Interleukin 8	KU_881637.1	F: GGGTGTAGATCCACGCTGTC<br>R: AGGGTGCAGTAGGGTCCAGA	58	167
TNF α	JN_412133.1	F: CTGGTGATGGTGTCGAGGAGGA<br>R: TCTGAGACTTGTTGAGCGTGAA	58	127
NOD	FJ_937972.1	F:ATGGTGGAAGAAGTCTGGCA<br>R: CTCGTTTTGTATTAGCATCAG	58	158
IRF 3	JQ_478481.1	F: GGAGACCACTCTGTTTGGAAG<br>R: CGGCATCGTTCTTGTTGTC	58	88
IRF 7	JQ_698666.1	F: TCCACTGAGGGTCTGATTGA<br>R: CGCTGGTGCTGACGAAGA	58	148
TLR 2	FJ_858800.2	F: GAACCTTGTAGGAAACCCAT<br>R:CCCATCTAAGCCATTCTTGT	58	127
TLR 3	DQ_885910.1	F: CTGTCTTCCTTGCTTTTGTACTCG<br>R: CCCAGTTTAGCAGATTTCAGTTTGT	58	290
TLR 7	AB_553573.1	F:GGGAATGCAATGAGCCAGA<br>R:GAAGAGAACATCAAATCCAGACGA	58	104
IFN a1	AB_376666.1	F: CAGAGTCAATGCTCCGCTTG<br>R: CAAGAAACCTCACCTGGTCCTC	58	178
IFN a2	AB_376667.1	F: GATGAAGGTGCCATTTCCAAG<br>R: CACTGTCGTTAGGTTCCATTGCTC	58	245

IFN a1S	EC_393381.1	F: AGCGGCCTTGAAACACGTTGGAATC<br>R: CCACTCATTTCCCGAAGCAGA	58	110
Hepcidin	KC_795559.1	F: GCATGCGTCTGCATCCTCC<br>R: CTGGTTCTCCTGTGGTGCTT	58	96
Muc2	MF_380420.1	F: GCATCAACCTGCCATTCC<br>R: CAGCACAGTCGTCCACCAAG	58	118
Muc13	MF_380418.1	F: TCTTTTGGTTGTGATCGTGGTG<br>R: GGATGAGGAGCTTCCCTTTTG	58	109

F: Forward primer; R: Reverse primer.
